# Will the Kaplan Fiber Complex Be the ‘‘New Anterolateral Ligament’’? Insights from Direct Surgical Exploration in the Context of ACL Injury and Reconstruction

**DOI:** 10.3390/jcm11164842

**Published:** 2022-08-18

**Authors:** Alberto Grassi, Silvio Caravelli, Massimiliano Mosca, Stefano Zaffagnini

**Affiliations:** II Clinic of Orthopaedics and Traumatology, IRCCS Istituto Ortopedico Rizzoli, 40136 Bologna, Italy

According to the most popular scientific literature database in 2022, nearly 20 papers mentioning the “Kaplan Fiber” complex have been published in the last 2 years, highlighting the role of this anatomical structure in the context of anterior cruciate ligament tear. This trend seems to delineate a similar pattern (but to a lower extent) to what happened in 2013 after the “first” description of the anterolateral ligament.

However, why this interest in Kaplan fibers? 

Firstly, the Kaplan fibers represent the attachments of the iliotibial band to the distal femur (Figure 1). First described by Kaplan in 1958, these fibers attach the iliotibial band (ITB) to the upper portion of the lateral distal femur [1]. Some anatomic studies have even characterized its anatomy, identifying two discrete bundles [2,3,4,5]: the proximal bundle that originates on the ITB and inserts just distal to the termination of the intermuscular septum, and the distal one that runs with a proximal–lateral to distal–medial oblique orientation, inserting into the femur just proximal to the superior lateral genicular artery [2]. The importance of this structure has been highlighted in a recent biomechanical study [6]; it showed that sectioning of the Kaplan fiber (KF) attachment of the ITB to the distal femur led to greater internal rotation of the tibia at higher flexion angles (30–90°) compared with sectioning of the anterolateral ligament. Moreover, it was demonstrated that the anterolateral ligament and the Kaplan fibers contribute to the restraint of the pivot shift and anterior tibial translation in the ACL-deficient knee.

For these reasons, studies of the clinical role of the Kaplan fibers and their injury are ongoing. Several very recent papers tried to identify the incidence of Kaplan fiber injury in the context of ACL rupture. To this end, Berthold et al. [7], through a well-performed Magnetic Resonance study with three independent reviewers, retrospectively identified a relevant number of patients with concomitant injury to the Kaplan fibers, confirming the results of similar studies [8,9]. Other authors have tried to correlate Kaplan fiber injury with laxity or concomitant injuries, with contrasting and inconclusive results [8,10]. However, among the limitations reported in studies with such a design, one of the most important is the lack of a clinical and surgical correlation. In this regard, we would like to share our unique experience in this field.

As pupils of Prof. Maurilio Marcacci from Rizzoli (Bologna, Italy), our gold standard for ACL reconstruction since 1993 has been the “over-the-top” technique with lateral plasty [11]; since then, thousands of ACL reconstructions using this technique have been performed [12]. One of the most peculiar features of the technique is the necessity for a lateral approach to prepare the over-the-top position. This is achieved via a 3–4 cm straight incision proximal to the lateral epicondyle, and via splitting of the posterior third of the iliotibial band (ITB); in this way, after carefully sectioning the intermuscular septum and performing a blunt dissection with a finger, we can gain access to the posterior capsule and complete our reconstruction. This surgical approach gives us the ability to systematically explore the surrounding extra-articular structures.

In our experience, we only note bleeding in the subcutaneous tissue superficial to the ITB in extremely rare cases of acute multi-ligamentous injuries; however, the ITB has never been found to be injured or stretched at this level proximal to the lateral epicondyle. After splitting the ITB, we rather frequently note bleeding around the Kaplan Fiber Complex in acute ACL injuries in athletes such as professional footballers (where ACL reconstruction is usually performed within 10–15 days), often with (minor) concomitant injuries such as grade I–II MCL or meniscal tears. In these cases, in our experience, it is not possible to identify discrete lesions, but there is general bleeding in the area with edematous tissues. It is not plausible for this bleeding to come from the intra-articular space due to capsular injuries, since we do not note water extravasation after arthroscopy; thus, it could represent some form of injury to the Kaplan fiber complex. Our empiric findings seem extremely consistent with the MRI pattern reported by Berthold et al. [7] Another important insight is that these abnormalities are not seen in cases of chronic ACL reconstruction, where the area does not present gross abnormalities. It is important to note that in their study, Berthold et al. only included patients with an MRI performed within 3 months from trauma, and we agree that in non-acute or sub-acute situations, it could be difficult to identify existing or previous Kaplan fiber injuries. If these injuries tend to heal, or the exact aspect of the chronic abnormalities in this area, such as their clinical implication, are not known, they are worthy of future investigation.

Of course, our insights are derived from direct empiric experiences, and are not supported by the systematic data collection and analysis in this regard. However, considering the high interest in this field, we are not opposed to designing a clinical study to objectify these findings and eventually correlate the pathoanatomic features with the clinical features.

In trying to delineate the clinical relevance of Kaplan fiber complex injuries, we have to keep in mind the knee anatomy in relation with the ACL injury mechanism. After performing many video analysis studies, especially on footballers [13,14,15,16], and tridimensional bone models of ACL injuries [17], we reached the conclusion that at the moment of ACL injury, a real “subluxation” with anterior translation, valgus deviation and tibial proximalization occurs. In this way, some form of capsular stretching and possible lesions seem obvious. The ITB, indeed, has a broad course from the hip to the Gerdy’s tubercle, and thus, it can easily adapt to stretching thanks to the stress dissipation along its length. However, the Kaplan fibers (which connect the deep portion of the distal ITB to the distal femur) have a shorter and peri-articular course; thus, they are exposed to a possible sudden change in length at the time of ACL injury and tibial “subluxation”. Moreover, due to its long course and its distant anatomical origin from the knee joint, the ITB changes its orientation with respect to the lateral epicondyle with knee flexion when the ITB slides posteriorly to it (possibly guided by the Kaplan fiber itself). At the time of ACL injury, the knee joint increases its flexion, from early degrees (near 10–20°) at the time of the foot strike and following ACL rupture to more than 30–40° after the ligamentous rupture [18]; thus, posterior sliding of the ITB is supposed. However, considering the abnormal anterior translation and proximalization of the tibia with respect to the femur, it is possible that the normal biomechanical behavior of the ITB is altered, disrupting the isometry of the Kaplan fibers. This is likely to cause their injury, without, however, damaging the whole ITB; instead, the ITB “adapts” its course according to the joint position. On several videos of ACL injuries in footballers, is it possible to note a small cutaneous depression between the anterior margin of the ITB and the patella, which could represent ITB displacement in the area where the ITB and subcutaneous layers have adhesions (this is an area where discrete separation of the fascial layers is not clear, based on surgical dissection experience).

To conclude, injury to the Kaplan fiber complex in the setting of ACL injuries and, broadly, the role of the whole ITB in the context of ACL rupture and reconstruction, are anything but “vintage” topics; rather, they should be investigated in a deeper manner with the use of both old and modern approaches. After decades of focus on intra-articular structures and the recent renewed interest in their peripheral structures—thanks to the hype surrounding the anterolateral ligament—it is time to widen our perspective; this includes (again) the ITB among the relevant structures in the context of ACL surgery.

## Figures and Tables

**Figure 1:**
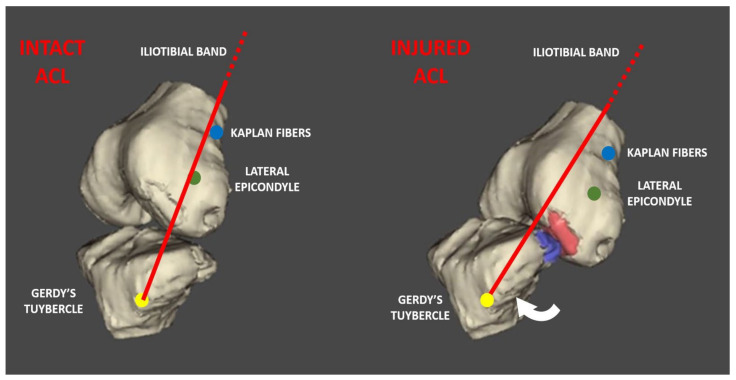
This 3D rendering shows the relationships between anatomical landmarks of the knee and the direction of the iliotibial band with an intact ACL and injured ACL.
